# Mobile-Assisted Cognitive Behavioral Therapy for Negative Symptoms: Open Single-Arm Trial With Schizophrenia Patients

**DOI:** 10.2196/24406

**Published:** 2020-12-01

**Authors:** Eric Granholm, Jason Holden, Kristen Dwyer, Tanya Mikhael, Peter Link, Colin Depp

**Affiliations:** 1 VA San Diego Healthcare System San Diego, CA United States; 2 Department of Psychiatry University of California, San Diego San Diego, CA United States

**Keywords:** motivation, persistent negative symptoms, dysfunctional attitudes, mHealth, blended intervention, mobile phone

## Abstract

**Background:**

Negative symptoms are an important unmet treatment need for schizophrenia. This study is a preliminary, open, single-arm trial of a novel hybrid intervention called mobile-assisted cognitive behavioral therapy for negative symptoms (mCBTn).

**Objective:**

The primary aim was to test whether mCBTn was feasible and could reduce severity of the target mechanism, defeatist performance attitudes, which are associated with experiential negative symptoms and poor functioning in schizophrenia.

**Methods:**

Participants with schizophrenia or schizoaffective disorder (N=31) who met prospective criteria for persistent negative symptoms were enrolled. The blended intervention combines weekly in-person group therapy with a smartphone app called CBT2go. The app extended therapy group skills, including recovery goal setting, thought challenging, scheduling of pleasurable activities and social interactions, and pleasure-savoring interventions to modify defeatist attitudes and improve experiential negative symptoms.

**Results:**

Retention was excellent (87% at 18 weeks), and severity of defeatist attitudes and experiential negative symptoms declined significantly in the mCBTn intervention with large effect sizes.

**Conclusions:**

The findings suggest that mCBTn is a feasible and potentially effective treatment for experiential negative symptoms, if confirmed in a larger randomized controlled trial. The findings also provide support for the defeatist attitude model of experiential negative symptoms and suggest that blended technology-supported interventions such as mCBTn can strengthen and shorten intensive psychosocial interventions for schizophrenia.

**Trial Registration:**

ClinicalTrials.gov NCT03179696; https://clinicaltrials.gov/ct2/show/NCT03179696

## Introduction

Negative symptoms account for much of the poor functional outcome in schizophrenia and are an unmet treatment need [1-3]. Negative symptoms can refer to reduced expressive (eg, facial affect and voice tone) or experiential (eg, avolition and asociality) symptoms, which comprise two separate factors [4-6]. Experiential negative symptoms are particularly important to treat, because they are strongly associated with functioning [5,7]. Unfortunately, available pharmacological treatments have only limited benefits for negative symptoms [8,9].

Beck and colleagues [10-12] have proposed that interventions that reduce defeatist attitudes may improve negative symptoms and functioning in schizophrenia. Several studies have found that cognitions such as defeatist performance (eg, “Why try, I always fail”) and social disinterest (eg, “I’m better off alone”) attitudes are associated with negative symptoms, and to some extent poor functioning, even after accounting for depression [10,13-18]. In social learning theory [19], self-competency beliefs are also central to motivation for achievement and engagement in effortful goal-directed activities. The Beck model hypothesis is that defeatist attitudes lead to low motivation and avoidance of effortful goal-directed activities. Thus, an intervention that targets defeatist attitudes may increase motivation and effort toward goal-directed activities.

Defeatist attitudes can be targeted in cognitive behavioral therapy (CBT). Clinical trials of CBT for psychosis have found mixed results for reducing negative symptoms [20,21], but some CBT interventions that specifically targeted defeatist attitudes have found more promising results [22-27]. Social skills training (SST) has also produced significant but modest improvements in negative symptoms [28,29]. In our cognitive behavioral social skills training (CBSST) intervention, which combines CBT and SST [30], we have found significant improvements in defeatist attitudes and in experiential negative symptoms and functioning; improvements in experiential negative symptoms were mediated by improvements in defeatist attitudes [23]. Modification of defeatist attitudes, therefore, may be a mechanism of change in CBSST, whereby reduction in defeatist attitudes contributes to increased motivation and effort toward goal-directed tasks. CBSST, however, is an intensive and lengthy (ie, up to 36 sessions), high-burden, multicomponent intervention. If the intervention could be shortened and the focus on reducing defeatist attitudes strengthened by using mobile interventions, the cost and burden of CBSST implementation could be reduced. In this way, blended interventions that combine mobile interventions with in-person interventions could increase access to evidence-based interventions for schizophrenia by reducing implementation barriers.

Smartphones are widely available, affordable, and frequently used by individuals with serious mental illness [31-34]. In previous clinical trials, we have found significant improvements in social functioning and symptoms in individuals with schizophrenia and bipolar disorder using 12-week mobile interventions that incorporate CBT principles with minimal therapist contact [35,36]. A number of other mobile interventions for schizophrenia have been developed and received preliminary testing, but few have specifically targeted negative symptoms or motivation and few have blended in-person plus app interventions; for reviews, see Camacho et al [37] and Firth and Torous [38]. One recent pilot trial of a blended intervention for psychosis [39] used a brief coping-focused intervention for distressing voices—SAVVy (Smartphone-Assisted coping-focused interVention for Voices)—which blended four sessions of in-person therapy with ecological momentary assessment (EMA) or ecological momentary intervention between sessions; the trial found that the intervention was feasible, improved coping, and, at a trend level, reduced severity of voices. Another app-only trial that did target motivation in schizophrenia—PRIME (Personalized Real-time Intervention for Motivational Enhancement) [40,41]—led to improvements in self-efficacy beliefs and social interactions with peers, as well as reduced defeatist beliefs and self-reported negative symptoms in individuals with recent-onset schizophrenia. These trials support the feasibility and potential benefits of this approach.

Given the promise of in-person and mobile CBT interventions targeting defeatist attitudes and motivation in schizophrenia, we developed a blended intervention, called mobile-assisted cognitive behavioral therapy for negative symptoms (mCBTn), which combines in-person, 90-minute, weekly groups with a mobile app called CBT2go. The mCBTn intervention primarily targets defeatist attitudes to improve experiential negative symptoms in schizophrenia. The CBT components and skills-training approach of our CBSST group intervention were combined with mobile thought-challenging interventions that were based on our Mobile Assessment and Treatment of Schizophrenia (MATS) [35] and CBT2go [36] interventions. This thought-challenging algorithm incorporated EMA to sample attitudes and moods in real-world contexts and then used personalized evidence to challenge dysfunctional beliefs (eg, if a participant rates their expectation for pleasure in a planned social interaction at a clubhouse as low, they would receive the challenge, “But you said you had fun at the clubhouse last time”). In addition to thought challenging, the CBT2go app and group intervention used in mCBTn both also incorporated recovery goal setting and tracking, scheduling pleasurable activities and social interactions, and pleasure-savoring interventions.

We conducted an open preliminary trial of mCBTn in patients with schizophrenia or schizoaffective disorder with moderate to severe persistent negative symptoms, and hypothesized that defeatist attitudes and experiential negative symptoms would be significantly reduced from baseline to end of treatment. This open trial was funded as part of the National Institute of Mental Health Experimental Therapeutics Program (RFA-MH-18-704 R61/R33), which involves preliminary testing of an intervention's impact on a target mechanism (ie, defeatist attitudes) associated with an important clinical outcome (ie, experiential negative symptoms). The primary aim of the study was target engagement; that is, we hypothesized that mCBTn would lead to a significant reduction in severity of defeatist attitudes. We also assessed participants at 12, 18, and 24 weeks of treatment to determine which dose of treatment could produce at least a medium effect size (Cohen *d*=0.5) improvement in defeatist attitudes. In the Experimental Therapeutics Program, contingent on changing the target in this single-arm open-trial phase, a larger randomized controlled trial (RCT) will be conducted with treatment at the dose identified.

## Methods

This was a single-arm, open-trial, pre-post evaluation of the feasibility and preliminary effect of mCBTn. This trial was registered at ClinicalTrials.gov (NCT03179696).

### Sample

The study protocol was reviewed and approved by the Institutional Review Board of the University of California, San Diego, prior to initiating research activities with participants. Participants with schizophrenia or schizoaffective disorder were selected who have moderate to severe persistent experiential negative symptoms [3], as well as moderate to severe defeatist attitudes, recognizing that an intervention targeting defeatist attitudes is not likely to be helpful for consumers who do not have them. Participants were required to meet all inclusion and exclusion criteria over a 2-week evaluation phase. Inclusion criteria were as follows:

1. Voluntary informed consent to participate and capacity to consent.
2. Aged 18-65 years.
3. Diagnostic and Statistical Manual of Mental Disorders, fifth edition (DSM-5), diagnosis of schizophrenia or schizoaffective disorder based on a Structured Clinical Interview for DSM-5 (SCID-5) interview and available medical record review.
4. Moderate to severe experiential negative symptoms in at least two of the three Clinical Assessment Interview for Negative Symptoms [5] Motivation and Pleasure (CAINS-MAP) domains (mean score of 2 [ie, moderate] or greater for items averaged within the Social, Work, or Recreational domain) at the beginning and end of the 2-week evaluation phase.
5. Moderate to severe defeatist attitudes (score of >50 on the Defeatist Performance Attitude Scale [DPAS] [10]).
6. A 6th grade or higher reading level on the Wide Range Achievement Test-4 Reading subtest.
7. Clinically stable and on stable medications (ie, no hospitalizations or medication changes in 4 months prior to enrollment).

Exclusion criteria were as follows:

1. Prior CBT in the past 2 years.
2. Greater than moderate Positive and Negative Syndrome Scale (PANSS) [42] positive symptoms (score >5 for any item: item P1 [delusions], item P2 [disorganization], item P3 [hallucinations], or item P6 [suspiciousness]).
3. Severe depression on the Calgary Depression Scale for Schizophrenia (CDS) [43] (score >8).
4. Extrapyramidal symptoms: Simpson-Angus Scale [44] (score >7).
5. DSM-5 alcohol or substance use disorder in past 3 months based on the SCID-5.
6. Level of care required interferes with outpatient therapy (eg, hospitalized or severe medical illness).
7. Unable to adequately see or manually manipulate the mobile device.

### Blended mCBTn Intervention

The therapy group and the mobile app integrated skills-based interventions, including recovery goal setting, thought challenging, scheduling of pleasurable activities and social interactions, and pleasure-savoring interventions to modify defeatist attitudes and improve motivation and pleasure negative symptoms. A modified 12-session version of the Cognitive Skills Module of CBSST [30] was delivered in 90-minute, weekly group therapy sessions with two masters-level therapists and approximately 6 participants per group. The 12-session module provided all the core mCBTn content within the minimum number of sessions that might be identified as the optimal dose, but the module was repeated for a total of 24 sessions to determine if more sessions were needed to change the defeatist attitude target.

The mCBTn manual included a therapist guide and a patient workbook describing the skills and homework assignments, as well as a collection of games and exercises to make learning fun and promote engagement [30]. The module began with setting a meaningful living, learning, working, or socializing recovery goal, and the goal was broken down into short-term goals and goal steps. Defeatist attitudes and avoidance behaviors that interfere with working on the goal were then modified using CBT skills. Group members were introduced to the general concepts of CBT, including the relationship between thoughts, actions, and feelings (ie, generic cognitive model); thought challenging through behavioral experiments and examining evidence for beliefs; and mistakes in thinking. The primary thought-challenging skill trained was the 3Cs: Catch It, Check It, Change It—“It” is an unhelpful defeatist thought. Group time was also spent practicing the smartphone interventions, developing content for the mobile device (eg, personalized motivational and thought-challenging statements about goals, socializing, and pleasurable activities), and reviewing data collected with the device to challenge defeatist attitudes.

An iPhone 5s or 5SE was provided to all participants with an unlimited data plan and could receive and send unlimited texts and phone calls. Mobile interactions were triggered by an app notification in the morning, with reminder prompts at midday and evening. If participants responded to the notification earlier in the day, the second and/or third notifications were not delivered. The purpose of the notifications was to prompt daily engagement with the app. Device training was provided on how to operate and charge the device, the meaning of all questions and response choices, procedures for carrying the device, responding to prompts, how to access crisis lines, and how to use various apps. This information was also provided in a written manual given to participants. Participants returned the device at the end of treatment.

**Figure 1:**
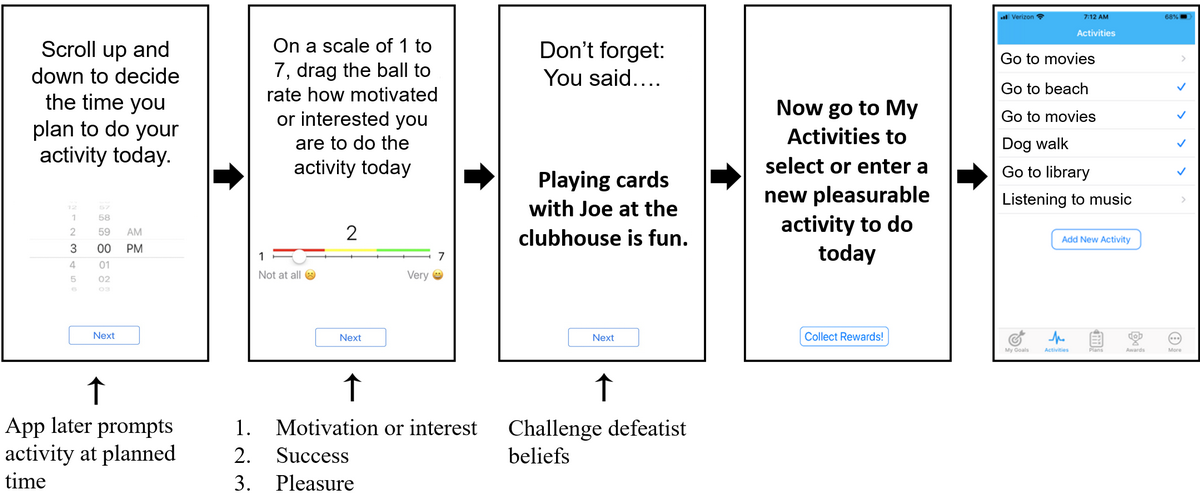
Screenshots of the CBT2go app for planning pleasurable or social activity. Low motivation, high defeatist attitudes (ie, anticipated success), or low anticipatory pleasure ratings triggered personalized reappraisal evidence. A separate My Activities tool was used to plan and savor activities.

The CBT2go app was used to prompt and track each group member’s goal-directed activities in the community, facilitate adherence to homework assignments involving community practice of thought-challenging skills trained in group, and prompt performance and savoring of personalized pleasurable activities and social interactions planned in group. The CBT2go app used personalized statements developed in group to challenge social disinterest and defeatist attitudes in real-time, real-world environments. After groups, therapists could enter personalized comments that participants made in group into a web-based dashboard (eg, “Having coffee with Jim is fun” and “Angie always makes you laugh”), which were used by the app to challenge low expectation ratings of motivation, anticipatory pleasure, or anticipated success for planned activities (see sample screenshots in Figure 1).

Participants carried the device and received the mobile intervention for the entire 24-week, blended group-plus-app intervention period. Each day during treatment, the CBT2go app alerted participants in the morning to make an *action plan*, which involved responding to a multiple-choice question about what to do that day: (1) a goal step, (2) social interaction, (3) pleasurable activity, or (4) homework assignment, or take the day off. Following the participant’s choice, the CBT2go app asked for three EMA ratings (slide bar ranging from 1 [not at all] to 7 [very much]) of (1) *motivation* to do the activity, (2) anticipated *success,* and (3) anticipated *pleasure* for the action plan. High ratings were reinforced (eg, “That’s right, socializing can be fun”), and low ratings were challenged (eg, “Don’t forget, you said walking on the beach was fun” or “But your goal is to make a close friend”). Behavioral experiments were then suggested to test out beliefs (eg, “Try asking someone to go for a short walk”). If a pleasurable activity action plan was selected, the pleasurable activities component of the CBT2go app was opened, which displayed several personalized activities that the participant previously entered. For the selected activity, the time and place to do the activity was queried (ie, a reminder alert was delivered at the time planned) and anticipated pleasure ratings were queried on a slide bar ranging from 0 (low) to 10 (high). Pleasure was again rated if the activity was completed, and these ratings were saved and available on demand in the group sessions for the therapist to discuss how people often think activities will not be as fun as they turn out to be and how this low expectation can reduce the likelihood of doing the activity. The app also prompts pleasure savoring of completed activities by taking a selfie or other photo or journaling about their experiences in the app. These photos and journal entries are also available on demand for review by participants or therapists in group sessions. Therapists helped develop the plans for pleasurable activities in group. Finally, if homework was the action plan selected, participants were directed to use their homework sheets and workbooks from the group to complete their weekly homework assignment.

Finally, an on-demand recovery goal–setting component of the CBT2go app was also provided and was populated during the goal-setting sessions in group by therapists and participants, as well as between sessions for homework by participants. A long-term goal was set and short-term goals and goal steps that would facilitate achievement of the long-term goal were entered into the app. The app could be accessed on demand to remind participants of goals, and goal steps could be checked off to track and motivate goal progress.

### Assessments

Participants were assessed at 2 weeks prior to baseline; at baseline; and at 12, 18, and 24 weeks of treatment. Dysfunctional attitudes were measured on the DPAS [10] (ie, primary target mechanism) and the Asocial Beliefs Scale (ABS) [45]. The CAINS-MAP [5] subscale was the primary negative symptom outcome measure. The CDS [43] and the PANSS [42] positive symptom subscale were used to assess secondary symptom outcome domains. Functioning was assessed on the Abbreviated Quality of Life Scale (A-QLS) [46] and the Social Functioning Scale (SFS) [47].

### Statistical Analyses

Mixed-effects regression models, utilizing HLM (hierarchical linear modeling) v6.08 (Scientific Software International), were estimated to predict each in-lab outcome assessment and mobile CBT2go app ratings of motivation, success, and anticipated pleasure for activities using time in weeks since baseline as a level-1 predictor. Paired-sample, 2-tailed *t* tests between baseline and each follow-up assessment tested whether significant change was found for each outcome at each assessment point to inform the dose of treatment that might achieve a significant improvement in DPAS and CAINS-MAP scores with at least a medium effect size (Cohen *d*=0.5).

## Results

### Sample

**Figure 2:**
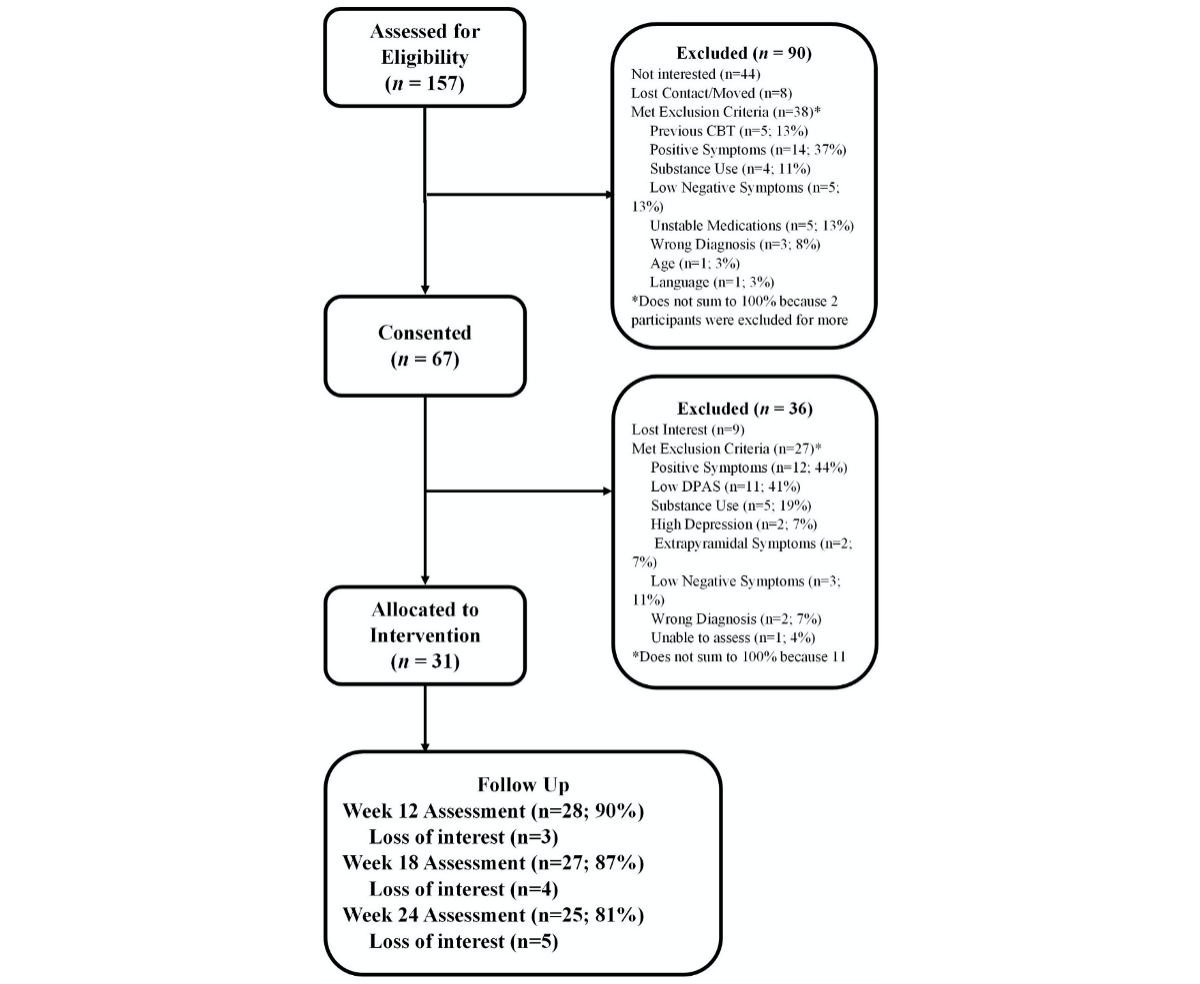
CONSORT (Consolidated Standards of Reporting Trials) flow diagram of participants through the open trial. CBT: cognitive behavioral therapy; DPAS: Defeatist Performance Attitude Scale.

We recruited and assessed 67 participants; see the CONSORT (Consolidated Standards of Reporting Trials) diagram in Figure 2. However, 36 of the 67 participants (54%) were excluded because they did not meet persistent negative symptom criteria. For the 31 participants enrolled in treatment, excellent retention was found with 28 (90%), 27 (87%), and 25 (81%) participants assessed at the 12-, 18-, and 24-week assessment points, respectively. There was only one adverse event, which was a hospitalization for symptom exacerbation in the context of medication nonadherence. The participant remained in the study. The sample had a mean age of 48.3 (SD 9.5) years and a mean of 11.8 (SD 1.5) years of education. The sample was 65% (20/31) male, was 65% (20/31) White, and had moderate baseline symptom severity—the PANSS total mean score was 60.5 (SD 12.7). Out of 31 participants, 21 (68%) had a schizophrenia diagnosis and 10 (32%) had schizoaffective disorder. Table 1 shows descriptive statistics and comparisons between baseline and each assessment point for all outcome variables.

**Table 1:** Outcome variables and paired *t* tests at each assessment point relative to baseline.

Outcome measure and time points		n (%)	Score, mean (SD)	Cohen *d*^a^	2-tailed *t* test (*df*)	*P* value
**Defeatist Performance Attitude Scale**						
	Baseline	31 (100)	66.3 (14.4)	N/A^b^	N/A	N/A
	12 Weeks	28 (90)	60.7 (15.9)	0.40	2.23 (27)	.03
	18 Weeks	27 (87)	56.4 (15.7)	0.70	4.02 (26)	<.001
	24 Weeks	25 (81)	52.2 (17.3)	1.00	4.10 (24)	<.001
**Asocial Beliefs Scale**						
	Baseline	30 (100)	5.9 (3.1)	N/A	N/A	N/A
	12 Weeks	28 (93)	6.5 (2.9)	–0.20	0.58 (27)	.57
	18 Weeks	27 (90)	6.2 (3.2)	–0.10	0.21 (26)	.84
	24 Weeks	25 (83)	6.4 (3.3)	–0.15	0.19 (24)	.85
**Clinical Assessment Interview for Negative Symptoms Motivation and Pleasure**						
	Baseline	31 (100)	23.1 (3.4)	N/A	N/A	N/A
	12 Weeks	28 (90)	21.3 (6.1)	0.55	2.07 (27)	.048
	18 Weeks	27 (87)	20.5 (6.2)	0.75	2.95 (26)	.007
	24 Weeks	25 (81)	20.0 (6.8)	0.90	3.16 (24)	.004
**Clinical Assessment Interview for Negative Symptoms Expression**						
	Baseline	31 (100)	4.9 (3.2)	N/A	N/A	N/A
	12 Weeks	28 (90)	4.7 (3.6)	0.05	0.56 (27)	.58
	18 Weeks	27 (87)	4.3 (3.3)	0.20	1.18 (26)	.25
	24 Weeks	25 (81)	4.4 (3.3)	0.15	1.11 (24)	.28
**Positive and Negative Syndrome Scale positive symptoms**						
	Baseline	31 (100)	13.4 (4.5)	N/A	N/A	N/A
	12 Weeks	28 (90)	13.0 (4.3)	0.10	0.75 (27)	.46
	18 Weeks	26 (84)	12.8 (4.8)	0.10	0.64 (25)	.52
	24 Weeks	24 (77)	11.5 (4.0)	0.45	2.46 (23)	.02
**Calgary Depression Scale**						
	Baseline	31 (100)	3.4 (2.2)	N/A	N/A	N/A
	12 Weeks	28 (90)	3.4 (2.6)	0	0 (27)	>.99
	18 Weeks	27 (87)	3.1 (2.5)	0.10	0.85 (26)	.40
	24 Weeks	25 (81)	2.7 (2.5)	0.30	1.99 (24)	.06
**Abbreviated Quality of Life Scale**						
	Baseline	31 (100)	23.3 (6.1)	N/A	N/A	N/A
	12 Weeks	28 (90)	25.8 (7.9)	0.40	2.17 (27)	.04
	18 Weeks	27 (87)	24.9 (8.3)	0.25	1.78 (26)	.09
	24 Weeks	25 (81)	24.8 (8.8)	0.25	1.77 (24)	.09
**Social Functioning Scale**						
	Baseline	31 (100)	114.7 (21.8)	N/A	N/A	N/A
	12 Weeks	27 (87)	118.5 (22.2)	0.15	2.27 (26)	.03
	18 Weeks	27 (87)	115.9 (23.6)	0.05	1.70 (26)	.10
	24 Weeks	25 (81)	117.3 (26.7)	0.10	1.70 (24)	.10

^a^A positive Cohen *d* value indicates improvement on the outcome from baseline to follow-up.

^b^N/A: not applicable.

### Dysfunctional Attitudes

The effect of time was significant for defeatist performance attitudes (DPAS: γ=–0.59, *t*_30_=–4.27, *P*<.001). Change in DPAS score from baseline (see Table 1) was significant at all assessment points during treatment, with medium to large effect sizes. The minimal treatment dose needed to achieve at least a medium effect size was at 18 weeks. In contrast, the effect of time was not significant for asocial beliefs (ABS: γ=0.01, *t*_29_=0.47, *P*=.64).

### Symptoms

Significant reduction in severity of experiential negative symptoms was found (CAINS-MAP: γ=–0.14, *t*_30_=–3.12, *P*=.004) with medium to large effect sizes (see Table 1). The minimal treatment dose needed to achieve at least a medium effect size was at 12 weeks. For expressive negative symptoms, assessed using the CAINS Expression (CAINS-EXP) subscale, the effect of time was not statistically significant and no significant reduction from baseline was found at any assessment point (CAINS-EXP: γ=–0.02, *t*_30_=–1.06, *P*=.30). Significant reduction in severity of positive symptoms was found by week 24, but not at earlier assessment points, and the effect of time was not significant (PANSS-positive subscale: γ=–0.06, *t*_30_=–1.46, *P*=.16). Similarly, significant reduction in severity of depressive symptoms was found by week 24 but not at earlier assessment points, and the effect of time was not significant (CDS: γ=–0.03, *t*_30_=–1.66, *P*=.11).

### Mobile Symptom and Attitude Ratings

CBT2go app ratings of motivation and anticipated pleasure and success for completing planned activities increased significantly for motivation (γ=0.007, *t*_28_=2.17, *P*=.04) and anticipated pleasure (γ=0.008, *t*_28_=2.53, *P*=.02) but not for anticipated success (γ=0.006, *t*_28_=1.62, *P*=.12). This indicates steady gains in self-reported motivation and pleasure (0.7-0.8 points per 100 days on a 0-7 scale) over treatment. To estimate effect sizes for these changes over the course of treatment, we computed the mean ratings for the first 4 weeks and last 4 weeks of treatment for mobile CBT2go app ratings. Large increases in mean ratings were found between the first 4 weeks of treatment and the last 4 weeks for motivation (Cohen *d*=0.65; first 4 weeks: mean 2.9, SD 1.8; last 4 weeks: mean 4.1, SD 1.8) and anticipated pleasure (Cohen *d*=0.80; first 4 weeks: mean 3.0, SD 1.5; last 4 weeks: mean 4.2, SD 2.1). Medium increases were found for anticipated success (Cohen *d*=0.55; first 4 weeks: mean 3.1, SD 1.5; last 4 weeks: mean 3.9, SD 1.9).

### Functioning

Significant improvement in A-QLS scores was found between baseline and 12 weeks but not at other assessment points, and the effect of time was not significant (γ=0.08, *t*_30_=1.49, *P*=.15). Participants also showed significant improvement on the SFS total score between baseline and 12 weeks but not at the other two assessment points, and the effect of time was only at a trend level (γ=0.19, *t*_30_=1.77, *P*=.09).

### App Engagement

The CBT2go app prompted participants to select an action plan each day during up to 168 days of treatment. There was a mean of 18.7 (SD 21.3) responses to 84 action plan prompts (22%) at 12 weeks and a mean of 32.3 (SD 31.5) responses to 168 action plan prompts (19.2%) at 24 weeks for participants who did not drop out of treatment by each assessment point; this indicates engagement in homework and skills practice more than once per week, with minimal fatigue effects over the course of treatment. The number of action plans completed was not significantly correlated with any symptom measure at baseline (range of *r*=–0.05 to 0.07).

In HLM analyses examining the association between app engagement and outcome, the number of action plans by time interaction was significant for change in CAINS-MAP scores (γ=–0.003, *t*_29_=–2.11, *P*=.04) but not for DPAS scores (γ=–0.002, *t*_29_=–0.36, *P*=.72). The change in CAINS-MAP scores relative to baseline was also marginally correlated with the number of action plans completed at 12 weeks (*r*=–0.33, *P*=.09) and 18 weeks (*r*=–0.35, *P*=.07) but not 24 weeks (*r*=–0.28, *P*=.18). This did not hold true for change in DPAS scores relative to baseline at 12 weeks (*r*=–0.23, *P*=.24), 18 weeks (*r*=–0.13, *P*=.51), and 24 weeks (*r*=–0.07, *P*=.75). Thus, greater engagement with the app was associated with greater reduction in motivation and pleasure negative symptoms.

## Discussion

The results of this open trial of mCBTn showed significant, large, within-group improvements in defeatist attitudes and negative symptoms and defeatist attitudes by 18 weeks of treatment, which demonstrates feasibility and engagement of the defeatist attitudes target, and justifies a larger RCT. These findings also provide support for the defeatist attitude model of negative symptoms [10,12]. Some specificity was found for improvements in defeatist performance beliefs but not asocial beliefs, perhaps because the intervention focused on all living, learning, working, and socializing goals, rather than only socialization. The intensive focus of mCBTn on modifying defeatist beliefs, both in group therapy and in real-world environments using the CBT2go app, resulted in a large improvement in experiential negative symptoms within a relatively short 18-week treatment duration and medium improvement by 12 weeks, which is relatively rapid change. If blended interventions such as mCBTn can strengthen and thereby shorten intensive psychosocial interventions, then implementation barriers associated with burden and cost of lengthy psychotherapy interventions would be reduced. This would improve access to evidence-based practices for consumers with severe and persistent negative symptoms, which would have considerable public health significance. However, further testing in a larger RCT with a control condition is needed to confirm the efficacy of mCBTn and justify work on implementation.

This study adds to the growing literature on CBT-based mobile interventions for schizophrenia [37,38,48,49] and indicates that patients with severe and persistent negative symptoms may be amenable to this approach. In the first trial to use mobile CBT-informed interventions for schizophrenia in a text-messaging platform, we [35] used a similar approach that integrated EMA of symptoms and behaviors (eg, social isolation, medication adherence, and voices) in everyday contexts and delivered personalized just-in-time thought-challenging messages when participants reported symptom distress or defeatist attitudes; we also found improvements in auditory hallucinations, socialization, and medication adherence. In another prior trial using this same algorithm in an earlier version of the CBT2go app, we found improvements in total symptoms in a large RCT of patients with schizophrenia and bipolar disorder [36]. Ben-Zeev and colleagues [50,51] also used a similar CBT-informed algorithm with the FOCUS app and added sleep interventions and on-demand educational components; they found reductions in several symptom domains in schizophrenia. Finally, Bucci et al [52] developed the Actissist app, which uses an algorithm based on the cognitive model of psychosis, and found large improvements in symptoms relative to symptom monitoring only in participants with early psychosis.

Related to this, recent meta-analyses have suggested that SST may be a more effective treatment for negative symptoms of schizophrenia than CBT [28,29]. The mCBTn intervention in this study did not include the SST or problem-solving components included in CBSST. Thus, while SST may be an effective treatment for negative symptoms, this trial suggests that the CBT components of CBSST targeting defeatist attitudes can improve experiential negative symptoms without SST.

With regard to secondary outcomes, modest improvements were found in positive symptoms and depression with a longer 24-week treatment period, which was not expected, given that participants were screened for severe positive symptoms and depression. Changes in functioning were mixed, with significant improvements found early in treatment but then dissipated with only trend-level improvements found overall on the A-QLS and SFS. The 24-week follow-up period may be too brief to expect meaningful changes in functioning.

This study had a high exclusion rate during the run-in period, with 36 out of 67 (54%) participants not meeting the strict, persistent, negative symptom entry criteria. A high screen failure rate during run-in periods is common in clinical trials with similar persistent negative symptom criteria. For example, a screen failure rate of 44% was found in a psychosocial trial using similar criteria, except DPAS [26], and this rate is slightly higher than in pharmaceutical trials with similar criteria [26,53]. The proportion of patients with documented persistent negative symptoms (46%) is consistent with other estimates [3] and suggests negative symptoms are a common treatment need in patients with schizophrenia. Clinical trials of participants with persistent negative symptoms are rare, so this study makes an important contribution by demonstrating improvements in experiential negative symptoms as a primary target rather than secondary improvements related to changes in positive or depressive symptoms. It is important to note that participants were also recruited specifically for persistent *experiential* negative symptoms, which are more strongly linked to functional impairments than expressive symptoms [5,7,54,55].

Retention rates were excellent (81%-90% across assessments), especially for this negative symptom population, suggesting the intervention is feasible. It may be important that transportation was provided to therapy groups, which likely facilitated retention and may be necessary to maintain engagement of this population, especially in a large county with limited public transportation where this study was conducted. We have found much better retention in CBSST trials when transportation was provided [56] than when it was not [57]. The CBT2go app may have also promoted engagement, for example, through daily reminders of personalized recovery goals. The mCBTn intervention focused on recovery goal work, which can improve motivation and promote engagement in psychiatric rehabilitation [58].

Engagement with the CBT2go app was mixed. The app was designed to promote engagement in recovery activities as often as every day. On average, however, participants responded to prompts to make an action plan for the day about one and a half times per week. While this proportion of days with completed action plans may seem low, practicing skills and completing homework assignments more than once per week is greater homework adherence than would be expected with CBT group therapy alone, where participants are typically expected to complete a single homework assignment per week. Homework adherence in CBT psychosocial interventions across multiple disorders is approximately 20%-56%. Thus, completing approximately one and a half action plans per week is better community engagement in recovery activities than might be expected in CBT therapy alone with one assignment per week. Greater engagement with the app was also associated with greater improvement in motivational negative symptoms and was unrelated to baseline severity of negative symptoms, suggesting the app played an important role in strengthening the treatment’s impact on this important outcome.

This trial had several limitations. First, as described above, further app development is needed to promote engagement (eg, simplified interface and rewards and feedback to motivate). In addition, patients with greater severity of defeatist attitudes were recruited, because this was the target mechanism, so the findings may not generalize to patients whose experiential negative symptoms may not be related to defeatist attitudes. Participants were also excluded for severe positive symptoms or depression, so findings may not generalize to these populations. Finally, and importantly, this was a preliminary open trial that did not control for the effects of time, therapist contact, trips out of the home to come to group, socialization with staff and other patients, and other nonspecific factors. The next step is to complete an RCT with a contact control condition, which we are currently conducting. If this ongoing RCT with the modified CBT2go app confirms the findings of this open trial, this would provide stronger support for the defeatist attitude model of experiential negative symptoms and suggest that blended interventions like mCBTn can strengthen and shorten intensive psychosocial interventions for negative symptoms in schizophrenia.

## Abbreviations

ABS: Asocial Beliefs Scale
A-QLS: Abbreviated Quality of Life Scale
CAINS-MAP: Clinical Assessment Interview for Negative Symptoms Motivation and Pleasure
CBSST: cognitive behavioral social skills training
CBT: cognitive behavioral therapy
CDS: Calgary Depression Scale for Schizophrenia
CONSORT: Consolidated Standards of Reporting Trials
DPAS: Defeatist Performance Attitude Scale
DSM-5: Diagnostic and Statistical Manual of Mental Disorders, fifth edition
EMA: ecological momentary assessment
HLM: hierarchical linear modeling
MATS: Mobile Assessment and Treatment of Schizophrenia
mCBTn: mobile-assisted cognitive behavioral therapy for negative symptoms
PANSS: Positive and Negative Syndrome Scale
PRIME: Personalized Real-time Intervention for Motivational Enhancement
RCT: randomized controlled trial
SAVVy: Smartphone-Assisted coping-focused interVention for Voices
SCID-5: Structured Clinical Interview for DSM-5
SFS: Social Functioning Scale
SST: social skills training
